# Evaluating the Effectiveness of Digital Social Robots in Reducing Loneliness Among Community-Dwelling Older Adults in Japan: Randomized Controlled Trial and Qualitative Analysis

**DOI:** 10.2196/74422

**Published:** 2025-10-22

**Authors:** Hiroshi Murayama, Mai Takase

**Affiliations:** 1 Research Team for Social Participation and Healthy Aging, Tokyo Metropolitan Institute for Geriatrics and Gerontology, 35-2 Sakae-cho, Itabashi-ku, Tokyo, 173-0015, Japan, 81 3-3964-3241

**Keywords:** social robots, loneliness, psychological well-being, randomized controlled trial, qualitative analysis

## Abstract

**Background:**

Most studies on interventions using social robots to reduce loneliness have been conducted in facilities in Western countries.

**Objective:**

This study evaluated the effectiveness of digital social robot interventions in reducing loneliness among community-dwelling older Japanese adults using a randomized controlled trial and qualitative analysis.

**Methods:**

Individuals aged ≥65 years who lived alone in Tokyo and neighboring areas and experienced loneliness were recruited. In total, 73 eligible participants were randomly assigned to either an intervention or a control group. The 4-week intervention involved a humanoid social communication robot (BOCCO emo), which facilitated conversations with human operators and family members and reminded participants of daily tasks. The primary outcome was loneliness, with mental health (psychological well-being, depression, and self-rated health), the frequency of laughter in daily life, health competence, and interpersonal relationships (social network and generalized trust) as secondary outcomes. Participants were evaluated at baseline and follow-up using a self-administered questionnaire. In the follow-up survey, participants in the intervention group provided open-ended responses regarding their experiences using the social robot.

**Results:**

In total, 68 participants completed both the baseline and follow-up surveys (34 in each group). The average age of the participants was 82.3 (SD 6.5) years, and 64 (N=68, 94%) participants were women. A linear mixed-effects model with a random intercept indicated that loneliness decreased more in the intervention group than in the control group (difference-in-difference −3.1, 95% CI −5.9 to −0.4). Psychological well-being also improved in the intervention group (difference-in-difference 1.9, 95% CI 0.1 to 3.7). We identified 4 categories through content analysis: *emotional support and psychological connection*, *lifestyle assistance*, *enrichment of social interaction*, and *cognitive and mental stimulation*.

**Conclusions:**

Social robots can reduce loneliness among community-dwelling older adults in non-Western societies. Information and communication technology appears to be an effective approach for alleviating loneliness and enhancing well-being among older adults in community settings.

## Introduction

Loneliness is an increasing public health concern, particularly among older adults [1]. The prevalence of loneliness is expected to increase as the global population ages, placing greater strain on health care systems [2,3]. Subjectively, loneliness arises when actual and desired social connections vary, leading to negative emotions such as anxiety, low self-esteem, and social distress [4]. Meta-analyses associate loneliness with an increased risk of mortality [5,6], cardiovascular and cerebrovascular diseases [6,7], mental disorders [6], and dementia [8]. Psychosocial interventions have been effective in reducing loneliness among older adults [2,9-12]. Masi et al [2] identified 4 key strategies for alleviating loneliness: (1) improving social skills, (2) enhancing social support, (3) increasing social contact, and (4) addressing maladaptive cognition. Online interventions have also demonstrated effectiveness [9,12], highlighting the potential value of technology-based approaches.

Moreover, artificial agents such as social robots may serve as effective tools for delivering loneliness interventions to older adults, offering social interaction and reciprocally responding to users [13]. Several randomized controlled trials (RCTs) have also assessed their impact [14]. Regarding loneliness, 3 RCTs have evaluated the use of social robots in care facilities. Banks et al [15] examined the effect of an artificial intelligence robot (AIBO) on loneliness in long-term care, whereas Robinson et al [16] evaluated the effect of a Paro robot in rest homes and hospitals; both studies reported reductions in loneliness. In contrast, Papadopoulos et al [17] tested the use of the Pepper robot in long-term care homes but found no significant changes. Yen et al [18] conducted a meta-analysis of RCTs and confirmed the effectiveness of social robots in reducing loneliness.

Current RCTs on social robot interventions for older adults can be improved in 2 ways. First, most studies have been conducted in care settings. Three of the RCTs included in the meta-analysis by Yen et al [18] focused on care facility residents. Thus, more rigorous RCTs are needed to assess effectiveness [14,18,19], particularly for community-dwelling older adults. This population’s more variable and less-structured social environments present unique methodological challenges that require a careful study design and rigorous control of confounding factors to accurately assess intervention effects. Second, most RCTs on health-related outcomes have been conducted in Western countries [14]. However, attitudes toward robots differ across cultures. Older Japanese individuals tend to anthropomorphize robots and form emotional bonds, creating a dynamic distinct from that observed among Western users [20]. Studies conducted in non-Western contexts would provide unique insights into the role of social robots in alleviating loneliness. Given the rapid aging of the population in many Asian countries, loneliness is likely to emerge as a pressing social challenge. Understanding the role of social robots in addressing loneliness in these regions is crucial for developing effective interventions.

Furthermore, qualitative research has been conducted to explore the impact of social robots on loneliness [21]. Such studies provide valuable insights into the underlying mechanisms by which social robots influence loneliness, offering perspectives that quantitative research alone may not be able to capture. By incorporating qualitative approaches, researchers can better understand the subjective experiences of users, the emotional responses, and the specific contexts in which social robots contribute to social well-being.

Therefore, in this study, we aimed to investigate the effectiveness of a digital social robot intervention in reducing loneliness among community-dwelling older Japanese individuals using both an RCT and a qualitative analysis.

## Methods

### Study Design and Participants

This RCT was conducted from February 2023 to June 2024. Data collection took place at participants’ homes, with evaluations conducted at 2 time points using a self-administered questionnaire: baseline (T1) and follow-up (T2). The follow-up survey collected qualitative data from the intervention group in an open-ended format.

Participants were recruited from Tokyo and neighboring regions through flyers at community centers and local events, including referrals from community comprehensive support centers and social welfare councils. The flyer invited older adults to “engage in daily communication and conversation using a robot,” with the aim of promoting natural interaction in everyday life. It did not explicitly emphasize loneliness reduction. The eligibility criteria were as follows: (1) aged ≥65 years; (2) living alone in the community; (3) experiencing loneliness (defined as a score of ≥6 on the University of California, Los Angeles [UCLA] Loneliness Scale, version 3, Short Form 3-item with a score range of 3‐12 [22,23], which may indicate probable depression [24]); and (4) capable of operating and interacting with the robot. Participants were excluded if they had cognitive or physical impairments, which were assessed based on the certification of the long-term care insurance system. Furthermore, as part of the eligibility assessment, research staff, who were not medical professionals, conducted home visits to observe the participants and evaluate the absence of cognitive and physical impairments.

Participants were randomly allocated to the intervention or control group using a computer-generated randomization sequence in a 1:1 ratio. Owing to the nature of the intervention, blinding participants and intervention providers was not feasible; however, the data analysts were blinded to the group allocation.

### Intervention

**Figure 1.**
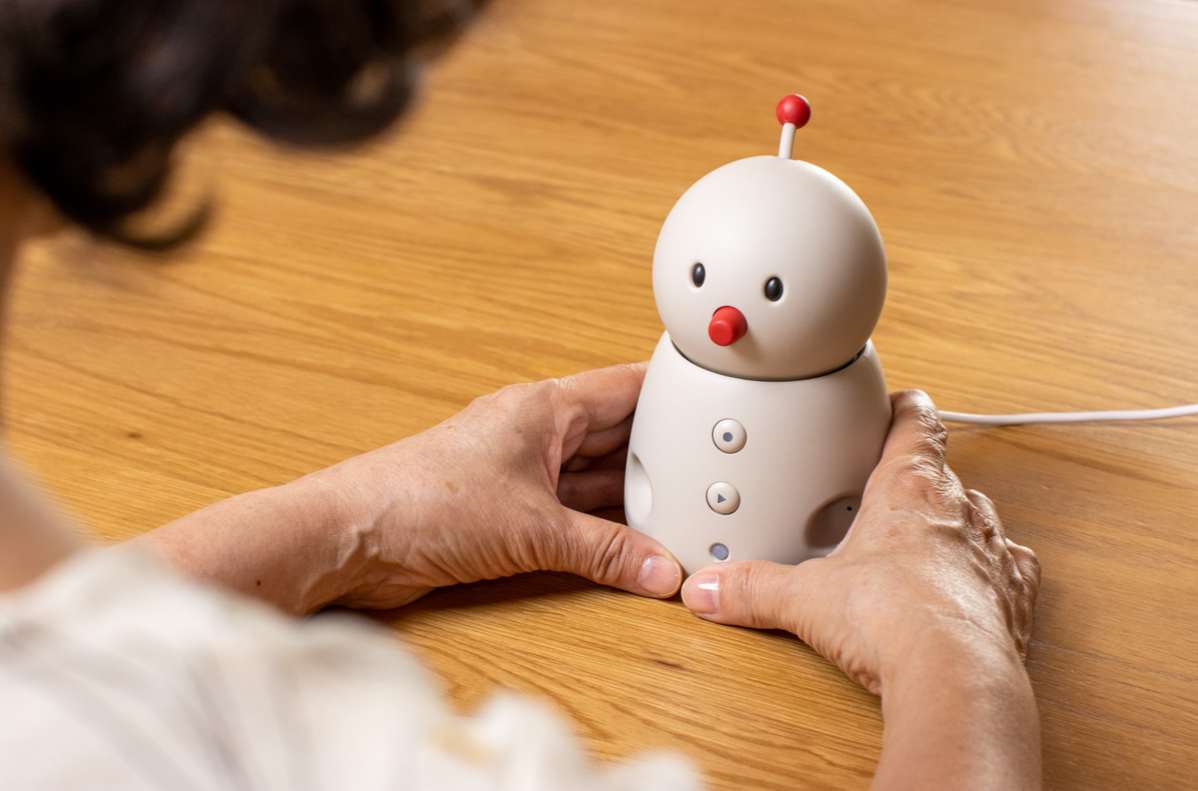
Representative image of BOCCO emo (Yukai Engineering Inc).

The intervention involved the use of a humanoid social communication robot, BOCCO emo (Yukai Engineering Inc; Figure 1), for 4 weeks. This service used a human operator–mediated system provided by SECOM Co, Ltd, and DeNA Co, Ltd, offering remote communication support. Before the intervention, the staff of SECOM visited the participants’ homes to set up BOCCO emo. At that time, they trained the participants on how to use BOCCO emo and provided information about the consultation service in case of problems.

Participants engaged with the robot by sharing daily experiences, thoughts, or concerns. These voice messages were transmitted to trained operators, who crafted empathetic and thoughtful responses tailored to each user. The responses were then vocalized by BOCCO emo, enabling participants to experience natural dialogue. This operator-mediated conversation system was available at all hours (the operators worked in shifts), allowing users to share their thoughts or concerns at any time, with the assurance of receiving a supportive response. A guide was prepared for operators to ensure quality in their service. As the operators worked in shifts, the operators who responded to the participants varied by day and time.

Morning and evening activities, including greenery-themed interactions, quizzes, and trivial tasks, were preprogrammed and automatically delivered through BOCCO emo. Following these automated messages, operators engaged in personalized interactions by responding to individual messages. In addition to operator interactions, family members could communicate with participants through BOCCO emo by entering messages via a smartphone, which were then vocalized by the robot. During the intervention period, the average number of message exchanges (ie, one back-and-forth between BOCCO emo and the participant) per participant was 5.5 (median 4.1, range 1‐18) times per day.

Beyond facilitating conversations, the intervention incorporated a reminder function to help participants manage essential daily tasks, such as taking medications, eating meals, and attending appointments. These reminders were preprogrammed and delivered through BOCCO emo in a friendly and accessible manner, supporting users in maintaining independence and adherence to routines.

Participants in the control group did not use social robots during the study period (any other service was not provided). However, to ensure fairness, they were offered the option to use BOCCO emo for 4 weeks after the study period concluded.

### Measures

Data collection before and after the intervention was conducted using a questionnaire, which was distributed when the staff of SECOM visited the participants’ homes to set up and pick up BOCCO emo before and after the intervention, respectively. The questionnaire was self-administered, and no external individuals were involved in the response process, reducing the risk of expectation bias.

#### Quantitative Data

The primary outcome was loneliness, assessed using the 20-item UCLA Loneliness Scale (version 3) [25]. The Japanese version of the scale was used, whose validity and reliability have been previously confirmed [26]. Respondents rated each item on a 4-point scale (“never,” “rarely,” “sometimes,” or “always”). Possible scores ranged from 20 to 80, with higher scores indicating greater loneliness. The Cronbach α values in this study were 0.91 at baseline and 0.93 at follow-up.

Secondary outcomes included mental health (psychological well-being, depression, and self-rated health); the frequency of laughter in daily life; health competence; and interpersonal relationships (social network and generalized trust). Psychological well-being was evaluated using the Japanese version of the World Health Organization-Five Well-Being Index [27,28]. This scale consists of 5 items assessing how respondents had felt over the past 2 weeks, rated on a 6-point scale (“at no time,” “some of the time,” “less than half of the time,” “more than half of the time,” “most of the time,” or “all of the time”). Scores ranged from 0 to 25, with higher scores indicating better psychological well-being. The Cronbach α values in this study were 0.79 at baseline and 0.83 at follow-up. Depression was measured using the 8-item Center for Epidemiologic Studies Depression Scale [29,30]. Respondents rated each item on a 4-point Likert scale (“rarely or none of the time,” “some or a little of the time [1‐2 days],” “occasionally or a moderate amount of time [3‐4 days],” or “all of the time [5‐7 days]”), with total scores ranging from 0 to 24. The Cronbach α values in this study were 0.80 at baseline and 0.84 at follow-up. Self-rated health was assessed by a single item (“How do you rate your overall health?”) with a 4-point scale (1=poor, 2=somewhat poor, 3=somewhat good, or 4=good).

The frequency of laughter in daily life was assessed as a behavioral outcome using a single self-reported item: “How often do you usually laugh in daily life over the past month?” Responses were recorded on an 8-point scale (1=rarely/none, 2=a few times, 3=once a week, 4=2‐3 times a week, 5=4‐5 times a week, 6=once a day, 7=a few times a day, or 8=many times a day). Although this outcome was not prespecified in the trial registration due to limited prior evidence on its relevance to the primary study objectives, we included it in the analysis as an exploratory variable. Given growing interest in the association between laughter and health [31,32], we report these results to enhance transparency and to guide future research.

Health competence was evaluated by the Perceived Health Competence Scale, Japanese version [33]. The scale consists of 8 items with a 5-point scale (“agree,” “somewhat agree,” “neither,” “somewhat disagree,” or “disagree”). The possible score range was from 5 to 40. The Cronbach α in this study was 0.86 at both baseline and follow-up.

The frequency of interaction with others was assessed using face-to-face or non–face-to-face interactions with relatives and friends, excluding cohabiting family members. Participants were asked four questions: (1) “How often do you meet or go out with friends or neighbors?” (2) “How often do you talk on the phone with friends and neighbors (including via email and fax)?” (3) “How often do you see or go out with family members or relatives you do not live with?” (4) “How often do you talk on the phone with family members or relatives you do not live with?” The possible answers were as follows: “6‐7 times a week (almost every day),” “4‐5 times a week,” “2‐3 times a week,” “once a week,” “2‐3 times a month,” “once a month,” “less than once a month,” or “never.” Following previous studies [34], the monthly total frequency was calculated. Generalized trust was measured by 1 item (“Generally speaking, most people can be trusted”). The response categories were 1=strongly disagree, 2=disagree, 3=somewhat disagree, 4=neither, 5=somewhat agree, 6=agree, or 7=strongly agree.

Age, sex, marital status, employment status, education level, subjective financial stability, and disease history were included as background characteristics. These variables were assessed at baseline, whereas primary and secondary outcomes were evaluated at both baseline and follow-up.

In addition to these variables, we also enquired about the participant’s satisfaction with the intervention as a part of process evaluation in the follow-up survey. The question stated, “How would you rate your overall satisfaction regarding the service provided through BOCCO emo?” Possible answers were “satisfied,” “somewhat satisfied,” “somewhat unsatisfied,” or “unsatisfied.”

#### Qualitative Data

During the follow-up evaluation, participants in the intervention group were asked about their experiences using BOCCO emo through the questionnaire. Responses were collected in an open-ended format.

### Data Analysis

#### Quantitative Data

In a previous RCT, the scores on the 20-item UCLA Loneliness Scale followed a normal distribution, with an SD of 10.0. The difference in scores between the intervention and control groups after the social robot intervention using the Paro robot was 7.0 [16]. Therefore, it was postulated that a mean score difference of 7.0 between the intervention and control groups on the 20-item UCLA Loneliness Scale, the primary outcome of this study, would be meaningful. Consequently, a sample size of 36 participants per group (72 participants in total) was calculated as sufficient to reject the null hypothesis (ie, the population means of the intervention and control groups were equal), with 80% power at a 5% significance level, assuming a 10% dropout rate.

For the evaluation of the intervention effects, a linear mixed-effects model with a random intercept was used to compare changes in all outcomes between groups over the intervention period. Group (intervention or control), time (T1 or T2), and their interaction were defined as fixed factors, and the participants were assumed as random factors. The effect size of the intervention (b) and its 95% CI were calculated as an estimate of the difference-in-difference (DID) in score changes between groups (change in the intervention group minus change in the control group). With regard to the process evaluation, the distribution of satisfaction with the intervention is shown. All analyses were performed using SPSS Statistics (version 29; IBM Corp).

#### Qualitative Data

The Krippendorff content analysis was applied to examine free-text responses from participants [35]. All responses were transcribed verbatim and anonymized. Two researchers (HM and MT) independently reviewed the narrative data to identify recurring themes and collaboratively developed a coding framework. Based on this framework, all responses were coded, with adjustments made as necessary. To ensure reliability, interrater agreement was assessed using Krippendorff α, based on the responses of 2 researchers (other than those who reviewed the data). Finally, the frequencies and contextual meanings of the identified categories were interpreted.

### Ethical Considerations

The study protocol was approved by the ethics committee of the Tokyo Metropolitan Institute for Geriatrics and Gerontology (R22-009) on August 12, 2022. The written informed consent was obtained from all participants. The trial was registered with the UMIN Clinical Trials Registry (UMIN000050644) and was conducted in accordance with the registered protocol. Each participant was assigned a unique study ID, and the researchers engaged in the analyses had access only to anonymized data. No financial or other compensation was provided to the participants.

## Results

**Figure 2.**
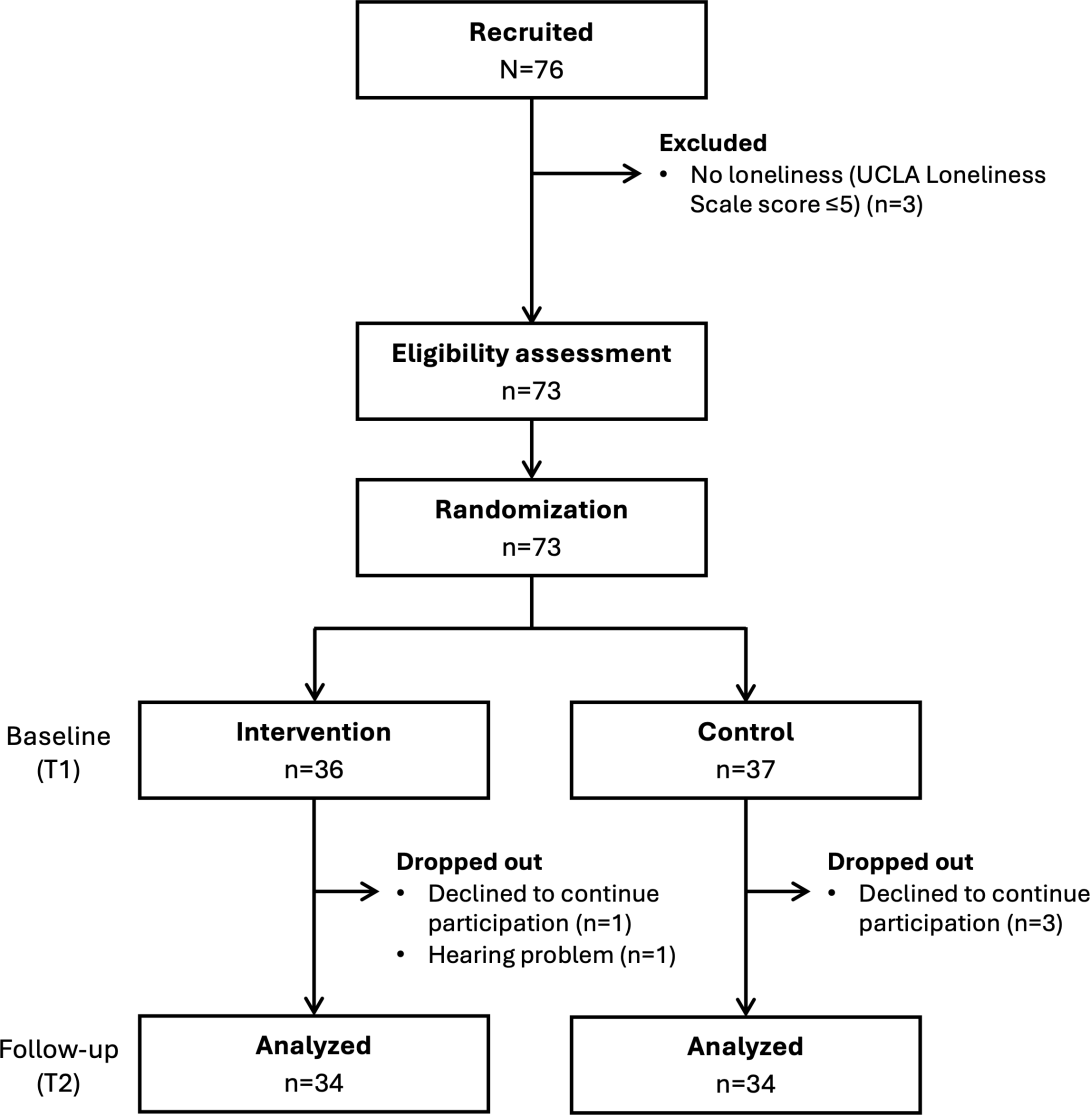
Flow diagram illustrating the selection process of study participants. UCLA: University of California, Los Angeles.

Figure 2 presents the flow diagram of the study participant selection for the 76 participants included in this study. All participants were aged ≥65 years, lived alone, and had not received certification for long-term care insurance (ie, they were physically and cognitively independent). Based on the 3-item UCLA Loneliness Scale, 3 individuals had scores of ≤5 and were excluded. In total, 73 individuals were deemed eligible. They were randomly assigned to the intervention (n=36) and control (n=37) groups. In the intervention and control groups, 5% (2/36) and 8% (3/37) of participants, respectively, dropped out of the study. Consequently, 68 participants who completed both the baseline and follow-up surveys were analyzed.

Table 1 summarizes the characteristics of participants in the intervention and control groups. The mean age was 82.3 (SD 6.5) years, and 64 out of 68 (94%) participants were women. More than 90% of the respondents (63 participants in each group) were unmarried or unemployed.

**Table 1.** Participants’ baseline characteristics (N=68).

Characteristics	Total	Intervention (n=34)	Control (n=34)
Age (y), mean (SD)	82.3 (6.5)	81.9 (6.8)	82.8 (6.2)
Gender, n (%)			
Woman	64 (94)	30 (88)	34 (100)
Marital status, n (%)			
Unmarried	63 (93)	30 (88)	33 (97)
Employment status, n (%)			
Unemployed	63 (93)	32 (94)	31 (91)
Education, n (%)			
Junior high school graduation	6 (9)	4 (12)	2 (6)
High school graduation	37 (54)	19 (56)	18 (53)
Junior college or vocational school graduation	10 (15)	5 (15)	5 (15)
University or college graduation	15 (22)	6 (18)	9 (27)
Subjective financial stability, n (%)			
Affluent	2 (3)	1 (3)	1 (3)
Somewhat affluent	20 (29)	10 (29)	10 (29)
Normal	40 (59)	18 (53)	22 (65)
Somewhat limited	5 (7)	4 (12)	1 (3)
Limited	1 (2)	1 (3)	0 (0)
History of diseases, n (%)			
Hypertension	32 (47)	15 (44)	17 (50)
Cancer	1 (2)	0 (0)	1 (3)
Heart disease	18 (27)	14 (41)	4 (12)
Stroke	3 (4)	2 (6)	1 (3)
Diabetes	9 (13)	5 (15)	4 (12)
Respiratory disease	5 (7)	3 (9)	2 (6)

Table 2 displays the scores and outcome changes between T1 and T2 for both groups. Regarding the primary outcome of loneliness, a significant effect of the intervention was observed. Changes in the loneliness score from T1 to T2 in the intervention and control groups were −3.7 and −0.6, respectively, with a DID value of −3.1 (95% CI −5.9 to −0.4; *P*=.03). In addition, participants in the intervention group exhibited a greater increase in psychological well-being than those in the control group, with a DID for the World Health Organization-Five Well-Being Index score of 1.9 (95% CI 0.1 to 3.7; *P*=.04). No significant difference in the DID of the other secondary outcomes was observed between the intervention and control groups. In terms of the process evaluation, 26 out of 34 (76%) participants in the intervention group reported being satisfied with the services through BOCCO emo (the data are not shown in the table).

Table 3 presents the content analysis results of feedback on the use of the social robot. Among the 34 participants in the intervention group, 42 codes were identified, leading to the classification of 4 categories. The Krippendorff α was 0.86, indicating high interrater reliability. The first category, *emotional support and psychological connection*, was central to participants’ experiences, as many found the robot reassuring and helpful in alleviating loneliness. Robot-mediated conversations fostered social and emotional well-being. The second category, *lifestyle assistance*, highlighted the role of the robot in managing daily routines. Participants reported that reminders and schedule management features helped maintain organization and stability. The third category, *enhancement of social interaction*, demonstrated the robot’s role in facilitating social engagement. It encouraged communication both with the robot and with other individuals, leading to more frequent social interactions. The fourth category, *cognitive and mental stimulation*, encompassed activities such as quizzes and information sharing. These interactions were perceived as beneficial for maintaining cognitive sharpness and promoting mental well-being.

**Table 2.** Score distribution and overall changes in the outcomes.

	Baseline (T1), mean (SD)	Follow-up (T2), mean (SD)	Difference (T2-T1), mean (SD)	Difference-in-difference (95% CI)	*P* value
Loneliness (UCLA^a^ Loneliness Scale; score range 20‐80)				−3.1 (−5.9 to −0.4)	.03
Intervention	41.2 (9.8)	37.4 (10.7)	−3.7 (6.3)		
Control	39.3 (12.4)	38.9 (11.1)	−0.6 (4.7)		
Psychological well-being (WHO-5^b^; score range 0‐25)				1.9 (0.1 to 3.7)	.04
Intervention	14.5 (3.9)	16.6 (5.4)	2.1 (4.4)		
Control	14.6 (6.6)	14.9 (6.3)	0.1 (3.0)		
Depression (CES-D-8^c^; score range 0‐24)				−1.3 (−3.3 to 0.7)	.21
Intervention	16.6 (3.5)	14.6 (5.0)	−2.0 (4.4)		
Control	16.6 (6.3)	15.9 (5.7)	−0.7 (3.9)		
Self-rated health (score range 1‐4)				0.0 (−0.3 to 0.3)	.89
Intervention	2.5 (0.9)	2.6 (0.9)	0.1 (0.7)		
Control	3.1 (0.9)	3.2 (0.8)	0.1 (0.4)		
Frequency of laughter (score range 1‐8)				0.5 (−0.2 to 1.1)	.18
Intervention	5.3 (1.9)	5.8 (1.6)	0.5 (1.3)		
Control	5.2 (2.0)	5.2 (1.8)	0.0 (1.5)		
Health competence (PHCS^d^; score range 8‐40)				0.0 (−2.6 to 2.5)	.98
Intervention	25.1 (7.4)	25.2 (5.9)	0.1 (6.7)		
Control	26.3 (8.3)	26.4 (9.1)	0.2 (3.2)		
Monthly frequency of contact with others except cohabitants				0.1 (−7.6 to 7.8)	.98
Intervention	36.6 (21.1)	36.8 (22.0)	0.2 (17.5)		
Control	37.3 (24.1)	37.4 (24.3)	0.1 (14.0)		
Generalized trust (score range 1‐7)				0.1 (−0.4 to 0.6)	.74
Intervention	4.7 (1.1)	4.6 (1.5)	0.0 (1.1)		
Control	4.9 (1.0)	4.8 (1.2)	−0.1 (1.0)		

a UCLA: University of California, Los Angeles.

b WHO-5: World Health Organization-Five Well-Being Index.

c CES-D-8: 8-item Center for Epidemiologic Studies Depression Scale.

d PHCS: Perceived Health Competence Scale.

**Table 3.** Content analysis of participants’ feedback on the social robot experience.

Category	Explanation	Codes, n	Example narratives
Emotional support and psychological connection	The robot’s presence and interactions provided psychological reassurance and a sense of connectedness.	17	“Just having it around made me feel calm as if I had a companion.” [Woman, aged 84 years] *“*Having recently lost my husband, the robot’s daily conversations were encouraging.” [Woman, aged 77 years] *“*Conversing with a robot can feel mechanical, but the conversation with BOCCO was similar to interpersonal communication, and that was reassuring.*”* [Woman, aged 90 years] *“*When I was called by my first name, it felt as if I had become younger.*”* [Woman, aged 83 years]
Lifestyle assistance	The robot assisted in schedule management and helped establish a structured daily routine.	9	“Even though I am aware of my schedule, it helped to be reminded.” [Man, aged 72 years] “The routine greetings at set times helped establish a good rhythm in my life.” [Man, aged 84 years]
Enrichment of social interaction	The robot facilitated social interaction and communication.	5	*“*I introduced BOCCO to my friends, and it became a topic of the conversation.” [Woman, aged 84 years] “I was happy to converse with my daughter and grandchildren through BOCCO.” [Woman, aged 89 years]
Cognitive and mental stimulation	The season-themed interactions, quizzes, and trivial tasks provided cognitive exercise and mental stimulation.	11	“The quizzes served as a cognitive exercise.” [Woman, aged 76 years]

## Discussion

### Principal Findings

To our knowledge, this is the first RCT to examine whether the intervention of a social robot reduces loneliness, specifically among older adults in Asian countries. This study is unique in that it integrates both quantitative and qualitative approaches. A qualitative approach is particularly useful for uncovering the mechanisms underlying the association between interventions and outcomes. As mentioned previously, cultural differences may influence attitudes toward robots. Recent reports indicate that loneliness increased worldwide during the COVID-19 pandemic. In Japan, older individuals experience more severe feelings of loneliness than other age groups [34]. Because most RCTs have been conducted in Western countries, a well-designed RCT in non-Western countries is necessary to provide valuable insights into how social robots can be tailored to meet the specific needs of older populations in diverse cultural settings.

This study found that in the intervention group, loneliness decreased while psychological well-being improved. Although previous RCTs have demonstrated that social robots significantly reduced loneliness among older adults [15,16], the effect size (DID) in this study was smaller. This may be because Japanese individuals tend to suppress negative emotions, such as loneliness, more than Westerners, as cultural norms prioritize social harmony over emotional expression [36]. Consequently, in the sample, the impact of robot intervention was modest. This study is noteworthy because it suggests that while social robotic interventions may be valuable, their effectiveness could be culturally dependent.

In addition to cultural factors, it is important to consider that this study targeted community-dwelling older adults rather than care facility residents, as in many previous studies. Care facility residents often experience more structured social environments with frequent interpersonal interactions, which may amplify the effects of social robot interventions. Conversely, community-dwelling older adults tend to live more independently, with greater variability in their social engagement and daily routines. These differences in living context could influence both the acceptability of robot interventions and their psychological impact. Therefore, the smaller effect size observed in this study may also reflect these differences in participants’ living situations rather than cultural factors alone. Future research should explore how social robot interventions can be tailored to the needs of community-dwelling older adults and maximize their benefits.

This qualitative analysis supports findings from a previous scoping review [21], which identified several key mechanisms through which social robots reduce loneliness and enhance well-being. Social robots provide emotional companionship, particularly for those living alone. Some studies have noted that older individuals felt the robot was waiting for them at home [37,38]. Indeed, the participants found its presence comforting and reported a reduction in a feeling of social isolation. Social robots also offer solace to those grieving the loss of a family member, similar to findings from previous research [39]. In addition, they foster engagement by relaying messages from human operators or family members, thereby reinforcing social ties. Their reminders helped users maintain routines and prevent disengagement, as observed in a German study [40]. These mechanisms alleviated loneliness and improved well-being by addressing emotional and social needs.

Furthermore, qualitative analysis revealed additional benefits. Social robots provide cognitive and mental stimulation through personalized conversations, quizzes, and knowledge sharing, enhancing engagement and emotional satisfaction. Participants noted that speech generated by human operators, rather than artificial intelligence, made the interactions feel authentic, thereby strengthening their psychological connections. While prior research has highlighted the role of social robots in remote communication, the findings suggest that these robots foster a sense of belonging by delivering personalized, emotionally supportive content. These insights expand existing frameworks, emphasizing cognitive and psychological engagement functions.

A previous meta-analysis on the effectiveness of social robots for depression [18] reported that the duration and style of intervention activities (individual or group-based) act as moderators, with longer interventions and group-based activities demonstrating greater effectiveness. Although this intervention lasted only 4 weeks and was conducted individually, it successfully reduced loneliness. This finding suggests that extending the duration or incorporating group sessions could further enhance the impact of the intervention. Future studies should explore these factors to optimize social robot interventions.

Unlike autonomous artificial intelligence–equipped communication robots, BOCCO emo operated through a human operator–mediated system. This distinctive feature may have contributed to participants perceiving their interactions as more humanlike rather than mechanical, which could have played a role in alleviating feelings of loneliness. Nonetheless, many of the participants’ experiences were similar to those reported in studies involving fully automated social or communication robots. Therefore, the findings from this RCT are likely to be generalizable to a broader range of social and communication robots.

Despite the neutral content of the recruitment materials, approximately 94% of participants in this study were women. This gender imbalance may reflect several factors, such as older women experiencing fewer psychological barriers to participating in communication-focused activities [41] and the demographic reality in Japan, where approximately 70% of older adults living alone are women [42]. As a result, our findings primarily reflect the effectiveness of communication robot interventions among older women. The effectiveness for older men remains unclear and should be examined in future research. The low participation rate for men may also suggest the need for alternative approaches tailored to their preferences, which is an important consideration when evaluating the external validity of our results.

This study had some limitations. First, because loneliness tends to be higher among older adults living alone in Japan [34], this population was targeted. Participants with higher levels of feeling lonely were included; thus, the findings reflect individuals with the potential for loneliness reduction and should be interpreted cautiously. Further research in the cohabiting populations is needed. Second, participants were not blinded to group allocation, which may have introduced expectation bias. However, when distributing the questionnaires, participants were explicitly asked to respond based on their current state and be as honest as possible. In addition, during the consent process, the study was described in neutral terms without emphasizing the expected outcomes to minimize potential bias. Third, information on the content of the conversations was not analyzed in this study due to personal information protection. However, qualitative analysis of the actual conversations through BOCCO emo would be useful for further understanding the mechanism underlying the intervention effects. Fourth, participant characteristics may have influenced the intervention effects, but this study did not account for these factors in detail. For example, loneliness levels are known to vary by marital status, such as being married, divorced, widowed, or never married [43]. In addition, this study did not collect data on participants’ family relationships or frequency of contact, which may also affect loneliness and the impact of the intervention. Although the RCT design helps mitigate the influence of confounding variables, the effects of these unmeasured factors cannot be ruled out. These limitations should be considered when interpreting the findings, and future studies should explore them more thoroughly. Finally, the trial was limited to Tokyo and neighboring regions. Generalizability to other community contexts requires further study as loneliness may vary by residential area characteristics [44].

### Conclusions

This study investigated the effectiveness of the digital social robot BOCCO emo in reducing loneliness among community-dwelling Japanese older adults who live alone. Most previous RCTs using social robots have been conducted in Western care facilities. Our findings indicate that social robots may serve as valuable tools for mitigating loneliness among community-dwelling individuals in non-Western populations. These results suggest that integrating information and communication technology into community settings could be an effective strategy for alleviating loneliness and enhancing the psychological well-being of older adults living in the community.

## Supplementary material

10.2196/74422 Checklist 1 CONSORT-eHEALTH checklist.

## Abbreviations

DID: difference-in-difference
RCT: randomized controlled trial
UCLA: University of California, Los Angeles
